# Efficient and Stable Subcellular Protein Labeling in *Leishmania mexicana* Using a Re-Engineered mNeonGreen Integration Vector

**DOI:** 10.3390/pathogens15040448

**Published:** 2026-04-21

**Authors:** Tianyu Lei, Mengtao Yu, Panjing Lv, Hui Deng, Di Yang, Kaijie Li, Yan Li

**Affiliations:** 1 Department of Pathogen Biology, School of Basic Medicine, Tongji Medical College, State Key Laboratory for Diagnosis and Treatment of Severe Zoonotic Infectious Diseases, Huazhong University of Science and Technology, Wuhan 430030, China; leitianyu@hust.edu.cn (T.L.); yumengtao303@163.com (M.Y.); panjinglv94@hust.edu.cn (P.L.); denghui1@hust.edu.cn (H.D.); d202481972@hust.edu.cn (D.Y.); 2 Hubei Provincial Center for Disease Control and Prevention, Wuhan 430079, China; 3 Pediatric Respiratory Disease Laboratory, Institute of Maternal and Child Health, Wuhan Children’s Hospital, Tongji Medical College, Huazhong University of Science and Technology, Wuhan 430014, China; 4 Hubei Provincial Key Laboratory of Pediatric Genetic Metabolic and Endocrine Rare Diseases, Wuhan 430030, China; 5 Department of Respiratory Medicine, Wuhan Children’s Hospital, Tongji Medical College, Huazhong University of Science and Technology, Wuhan 430014, China

**Keywords:** *Leishmania mexicana*, mNeonGreen, protein localization, flagellum, bioimaging

## Abstract

The protozoan parasite *Leishmania mexicana* serves as a widely used model for studying trypanosomatid biology, yet the demand for stable, high-intensity fluorescent tools for precise subcellular protein localization remains unmet. In this study, we developed a versatile molecular toolbox by re-engineering the *pLEXSY-hyg2.1* vector to express mNeonGreen (mNG), a next-generation fluorophore with superior brightness and photostability. Using a modular cloning strategy, we introduced a customized multiple cloning site (MCS) upstream of the mNG sequence to facilitate seamless N-terminal tagging of target proteins. The construct was integrated into the 18S rRNA locus via homologous recombination to ensure stable, constitutive expression. As a proof-of-concept, we fused a flagellar marker to the mNG reporter, resulting in a transgenic line exhibiting robust and specific subcellular fluorescence without compromising cellular fitness. Our results demonstrate that this integration-based system provides a highly efficient and stable platform for visualizing protein distribution within *Leishmania*. This tool significantly simplifies the generation of reporter strains and will facilitate high-resolution imaging studies of parasite organelle dynamics and functional genomics.

## 1. Introduction

*Leishmania* species are devastating protozoan parasites responsible for leishmaniasis, a spectrum of diseases that remains a major global health burden. In this study, *Leishmania mexicana* (*L. mexicana*) was selected as the primary model for methodological development due to its robust growth characteristics, well-defined developmental stages, and high amenability to genetic manipulation. As a widely recognized fundamental model, *L. mexicana* is frequently employed to investigate parasite development, host–pathogen interactions, and organelle biogenesis. By utilizing this well-characterized species, we aimed to establish a standardized molecular framework that can serve as a technical reference for future labeling strategies in other *Leishmania* species [1,2,3,4]. Central to these studies is the ability to accurately visualize protein localization and dynamics within the cell [5,6]. While various fluorescent labeling techniques have been developed for trypanosomatids, there remains a critical demand for reporters that combine extreme photostability with high signal-to-noise ratios to facilitate advanced imaging modalities, such as long-term intravital tracking and super-resolution microscopy [7,8,9].

Traditional episomal expression vectors in *Leishmania* often suffer from plasmid loss or significant cell-to-cell variation in expression levels under drug selection [10]. To overcome these limitations, genomic integration into high-copy-number loci, such as the 18S ribosomal RNA (rDNA) locus, has become a preferred strategy [11,12]. This approach ensures stable, constitutive expression of the transgene throughout the parasite’s life cycle. Furthermore, the choice of fluorophore is critical; while eGFP has been a workhorse for decades, its limitations in brightness and rapid photobleaching often hinder the observation of delicate subcellular structures or long-term parasite persistence in vivo. Next-generation proteins like mNG offer substantially higher brightness and superior photostability, which are not merely incremental improvements but essential requirements for high-contrast imaging in autofluorescent host environments [13]. Although endogenous tagging reflects physiological levels, it often fails to provide sufficient signal for high-speed or deep-tissue imaging. Therefore, genomic integration into the high-copy 18S rDNA locus remains a robust strategy to achieve the stable, high-intensity labeling required for functional tools and drug efficacy studies [14,15,16]. Moreover, the high cost and limited availability of specialized antibodies for *Leishmania* proteins often render immunofluorescence assays (IFA) prohibitively expensive and technically challenging, further underscoring the necessity for robust endogenous fluorescent tagging systems [7,17,18].

In this study, we describe the development of an optimized molecular toolbox for *L. mexicana* based on the *pLEXSY-hyg2.1* framework [19]. We re-engineered the vector to express an N-terminal mNG-tagging cassette, incorporating a modular multiple cloning site (MCS) to facilitate the rapid and seamless fusion of target proteins. By targeting the 18S rRNA locus, we achieved robust and uniform expression of mNG-fusion proteins, demonstrating that this system maintains high fluorescence stability without compromising parasite fitness. As a demonstration of the system’s utility, we successfully generated a transgenic line expressing a flagellar protein fused to mNG, which provided high-contrast, specific labeling of the flagellum. This versatile system simplifies the construction of reporter strains and provides a powerful platform for functional genomics and subcellular localization studies in *Leishmania*.

## 2. Materials and Methods

### 2.1. Leishmania Strains and Culture Conditions

The *L. mexicana* strain MNYC/BZ/62/M379 was used throughout this study. Promastigotes were cultured at 27 °C in M199 medium (Gibco, Grand Island, NY, USA) supplemented with 10% heat-inactivated fetal bovine serum (FBS; Sigma-Aldrich, St. Louis, MO, USA), 40 mM HEPES (pH 7.4; Solarbio, Beijing, China), 0.1 mM adenine (Sigma-Aldrich, St. Louis, MO, USA), 5 mg/L hemin (Sigma-Aldrich, St. Louis, MO, USA), 1 mg/L biotin (Sigma-Aldrich, St. Louis, MO, USA), and 1 mg/L biopterin (Sigma-Aldrich, St. Louis, MO, USA). To prevent bacterial contamination, the medium was supplemented with 50 U/mL penicillin and 50 μg/mL streptomycin (Hyclone, Logan, UT, USA). Parasites were maintained in the logarithmic growth phase by subculturing once a week at a dilution ratio of 1:20 to 1:40 into fresh medium.

### 2.2. Validation via PFR2-mNG Reporter Construction

To validate the utility of our tagging platform for subcellular imaging, we targeted the Paraflagellar Rod Protein 2 (PFR2, LmxM.16.1430) [6], a well-characterized structural component of the trypanosomatid flagellum. The full-length PFR2 coding sequence was amplified from *L. mexicana* genomic DNA. Using a Gibson Assembly Master Mix (New England Biolabs, Ipswich, MA, USA), the PFR2 amplicon was precisely inserted into the linearized MCS of Plasmid 1, upstream of the mNeonGreen (mNG) reporter. This strategy ensured the generation of an in-frame PFR2-mNG-Flag fusion construct under the control of the endogenous-like processing signals of the pLEXSY system. The fidelity of the resulting reporter (Plasmid 2) and the integrity of the fusion junction were rigorously confirmed by multi-locus restriction mapping and Sanger sequencing (Sangon Biotech, Shanghai, China).

### 2.3. Stable Transformation of L. mexicana

To generate stable transgenic lines, logarithmic-phase *L. mexicana* promastigotes were harvested by centrifugation (1500× *g*, 5 min) and resuspended in Tb-BSF buffer (90 mM Na_2_HPO_4_, 5 mM KCl, 0.15 mM CaCl_2_, and 50 mM HEPES, pH 7.3) to a final density of 1 × 10^8^ cells/mL. For genomic integration, 10–20 μg of the SwaI-linearized (New England Biolabs, Ipswich, MA, USA) PFR2-mNG-Flag construct was mixed with 400 μL of the cell suspension in a 2 mm gap cuvette, followed by electroporation using a BTX Gemini system (BTX, Holliston, MA, USA) with a multi-pulse protocol consisting of three consecutive pulses at 800 V and a 500 ms interval. Following a 24 h recovery period in drug-free M199 medium at 27 °C, selective pressure was applied with 50 μg/mL Hygromycin B. Stable transgenic populations typically emerged within 10–14 days and were directly utilized for subsequent high-resolution imaging and functional characterization.

### 2.4. RNA Extraction and Quantitative Real-Time PCR

Total RNA was isolated from *L. mexicana* promastigotes in the mid-logarithmic growth phase using the Total RNA Extraction Kit (Servicebio, Wuhan, China). First-strand cDNA was synthesized from 1 μg of total RNA using the HiScript III RT SuperMix (Vazyme, Nanjing, China) according to the manufacturer’s instructions. Quantitative real-time PCR (RT-qPCR) was performed on a QuantStudio Real-Time PCR System (Thermo Fisher Scientific, Waltham, MA, USA) using 2× SYBR Green qPCR Master Mix (Servicebio, Wuhan, China). The transcript levels of PFR2-mNG were normalized to the endogenous *GAPDH* (*LmxM.30.2470*) as an internal control. Relative gene expression was calculated using the 2^−(∆∆Ct)^ method. Data are presented as the mean ± SEM from four independent biological replicates.

### 2.5. Live-Cell Imaging and Fluorescence Stability Assessment

To evaluate the long-term genetic stability and fluorescence persistence of the mNG-tagged lines, *L. mexicana-mNG* promastigotes were subjected to longitudinal imaging over a continuous 9-week period. For each weekly imaging session, mid-log-phase parasites were harvested and incubated with Hoechst 33342 (1 μg/mL) for 15 min at 27 °C to stain nuclear and kinetoplast DNA. Following two washes with PBS (1500× *g*, 5 min), the parasites were resuspended in 2% (*w*/*v*) methylcellulose in PBS to increase medium viscosity and minimize flagellar-driven motility.

A small volume (approximately 5–10 μL) of the cell suspension was applied onto a glass slide and covered with a high-precision coverslip (No. 1.5). The edges were sealed to prevent evaporation during image acquisition. Samples were visualized using an Olympus confocal laser scanning microscope (Olympus Corporation, Tokyo, Japan) equipped with a 20× or 100× oil immersion objective. mNG fluorescence was excited at 488 nm, and Hoechst 33342 (Thermo Fisher Scientific, Waltham, MA, USA) was excited at 405 nm. To ensure quantitative comparability across the 9-week duration, all images were acquired using identical laser power, gain, and offset settings. The maintenance of robust fluorescence signals throughout the observation period confirmed the successful integration and stable expression of the reporter construct at the 18S rRNA locus.

### 2.6. Flow Cytometry Analysis for Fluorescence Stability

To quantitatively evaluate the long-term genetic stability and fluorescence intensity of the mNG reporter, *L. mexicana-mNG* promastigotes were subjected to longitudinal flow cytometry analysis over a continuous 7-week period. For each weekly measurement, mid-log-phase parasites were harvested, washed twice with PBS, and resuspended at a density of 1 × 10^6^ cells/mL. Data acquisition was performed using a DxFLEX flow cytometer (Beckman Coulter, Brea, CA, USA). The mNG fluorescence was excited using a 488 nm laser and detected in the FITC channel (525/40 nm bandpass filter). For each sample, 5000 events were collected within the gated population. Parasites were identified based on their forward scatter (FSC) and side scatter (SSC) profiles to exclude debris and aggregates. The stability of the reporter was assessed by monitoring the percentage of mNG-positive cells and the mean fluorescence intensity (MFI) throughout the study. All data were analyzed using CytExpert or FlowJo software (Version 2.4, Beckman Coulter, Brea, CA, USA), with identical gain and voltage settings maintained to ensure quantitative comparability.

### 2.7. Macrophage Infection and Live-Cell Imaging

To observe the intracellular status of the parasites, the murine macrophage cell line RAW 264.7 was seeded into 35 mm glass-bottom dishes and cultured in DMEM supplemented with 10% FBS until they reached 70–80% confluence. The macrophages were subsequently challenged with stationary-phase *L. mexicana-mNG* promastigotes at a multiplicity of infection (MOI) of 10:1. Following 24 h of incubation at 37 °C, the infected monolayers were washed once with PBS to remove non-internalized parasites. Live-cell imaging was performed directly in the glass-bottom dishes using an Olympus confocal laser scanning microscope equipped with a 100× oil immersion objective. The mNG fluorescence was excited at 488 nm. Brightfield (BF) microscopy was employed simultaneously to define the macrophage boundaries and visualize the relative localization of the mNG-tagged parasites within the host cells.

### 2.8. In Vivo Infectivity and Ear Tissue Imaging

To evaluate the in vivo infectivity and reporter stability, female BALB/c mice (6–8 weeks old, *n* = 6) were utilized for a murine cutaneous leishmaniasis model. Mice were inoculated intradermally (i.d.) in the ear pinna with 1 × 10^6^ stationary-phase (or metacyclic) *L. mexicana-mNG* promastigotes resuspended in 10 μL of PBS. At 28 days post-infection (dpi), infected ears were harvested and embedded in OCT compound for cryosectioning. Tissues were processed into 30 μm sections at −20 °C and examined via fluorescence microscopy to visualize parasite distribution and signal persistence. This experiment was conducted in independent replicates to ensure reproducibility.

### 2.9. Parasite Burden Quantification via qPCR

To quantify the parasite load in the ear tissues, total genomic DNA was extracted using the DNeasy-based tissue DNA kit (Omega Bio-tek, Norcross, GA, USA) according to the manufacturer’s instructions. The concentration of the extracted DNA was determined using a spectrophotometer (Thermo Fisher Scientific, Waltham, MA, USA) to ensure consistency in the template input. The parasite burden was measured by quantitative real-time PCR (qPCR) targeting the Heat Shock Protein 70 (Hsp70) gene of *Leishmania*. The qPCR reactions were performed on a Thermo Fisher Scientific real-time PCR system (Thermo Fisher Scientific, Waltham, MA, USA) using 2× SYBR Green qPCR Master Mix (Servicebio, Wuhan, China). Absolute quantification was achieved using a standard curve generated from 10-fold serial dilutions of a plasmid containing the *Leishmania* Hsp70 sequence (10^1^ to 10^7^ copies). The final parasite load was expressed as the number of Hsp70 copies per mg of total tissue.

## 3. Results

### 3.1. Generation of an Ssu-Targeted mNG Integration Vector and Establishment of an mNG Reporter Line

**Figure 1:**
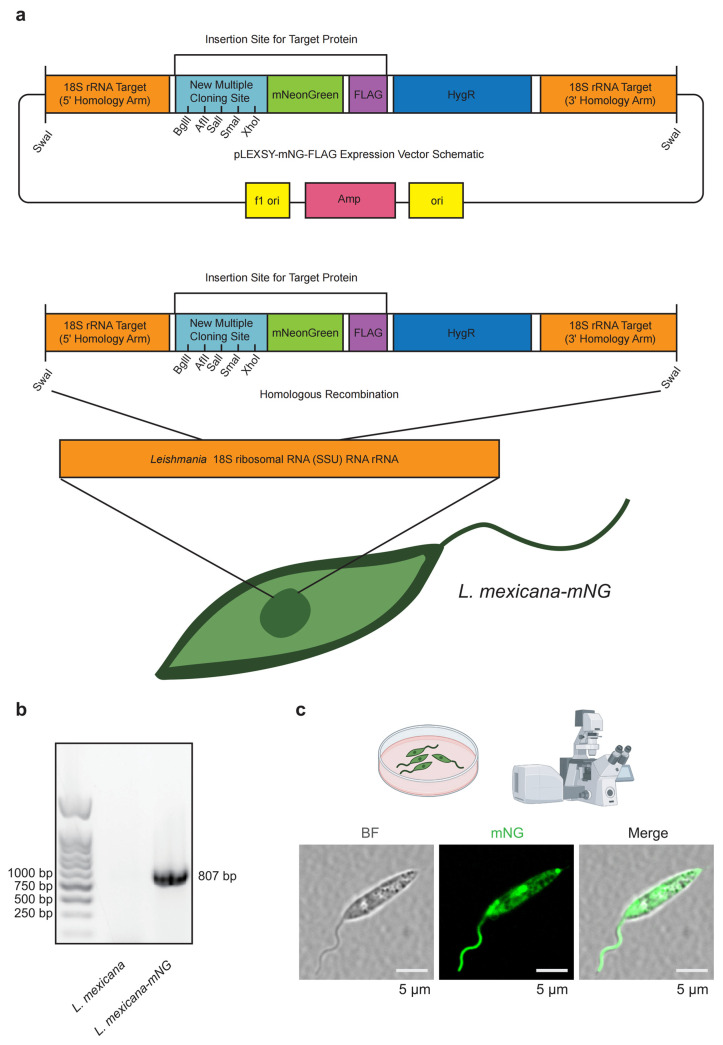
Generation and molecular validation of the *L. mexicana-mNG* reporter strain. (**a**) The pLEXSY-mNG-FLAG construct contains an mNG-FLAG fusion cassette, flanked by 5′ and 3′ homology arms (HAs) targeting the 18S rRNA locus. Following SwaI linearization, the cassette was integrated into the *L. mexicana* genome by homologous recombination. The construct also carries a hygromycin resistance marker (HygR) for selection. (**b**) Diagnostic PCR confirming site-specific integration. Genomic DNA from wild-type (WT) and *L. mexicana-mNG* parasites was analyzed using integration-specific primers. An amplicon of the expected size (807 bp) was detected exclusively in the transgenic line. Molecular weight markers (bp) are shown on the left. (**c**) Subcellular localization of mNG in *L. mexicana* promastigotes. Representative confocal images of mid-log-phase parasites show mNG fluorescence distributed throughout the cytoplasm and along the full length of the flagellum. Brightfield (BF) and merged images indicate preserved cell morphology. Images were acquired using an Olympus confocal microscope with a 100× oil-immersion objective. Scale bars, 5 μm. Images are representative of at least three independent experiments.

To facilitate the longitudinal tracking of *L. mexicana* both in vitro and in vivo, we engineered a transgenic reporter line expressing the high-intensity fluorophore mNG. We designed a *pLEXSY-mNG-FLAG* expression system (Figure 1a) where the mNG-FLAG cassette is flanked by homology arms (HAs) specific to the ribosomal RNA (rRNA) 18S locus. Following linearization with SwaI, the construct was integrated into the host genome through homologous recombination (HR) (Figure 1a). The 18S rRNA locus was selected as the integration site to ensure robust, constitutive expression of the reporter due to the high transcriptional activity and multicopy nature of this locus in *Leishmania*.

The fidelity of the genetic modification was validated by diagnostic PCR analysis of the genomic DNA. Using a specific primer set designed to span the integration junction, we amplified an 807 bp fragment from the *L. mexicana-mNG* candidate clones, whereas no product was detected in the parental wild-type (WT) DNA (Figure 1b). This binary result unequivocally confirms the successful site-specific insertion of the mNG reporter into the target 18S rRNA locus.

To assess the expression pattern and subcellular localization of the mNG reporter, mid-log-phase promastigotes were examined by confocal laser scanning microscopy. The mNG signal was observed as a strong and homogeneous fluorescence throughout the cytoplasm and extended along the entire length of the flagellum, enabling clear visualization of the flagellar axoneme (Figure 1c). Brightfield imaging and merged analyses showed that *L. mexicana-mNG* cells retained the characteristic spindle-shaped morphology and normal flagellar length, with no overt morphological abnormalities detected. Together, these results indicate that genomic integration of the mNG-FLAG cassette does not cause detectable perturbations to parasite morphology or development, supporting the use of this reporter line for downstream functional and cell biological studies.

### 3.2. Bright and Stable mNG Expression Across Parasite Stages and During Prolonged Culture Without Selection

**Figure 2:**
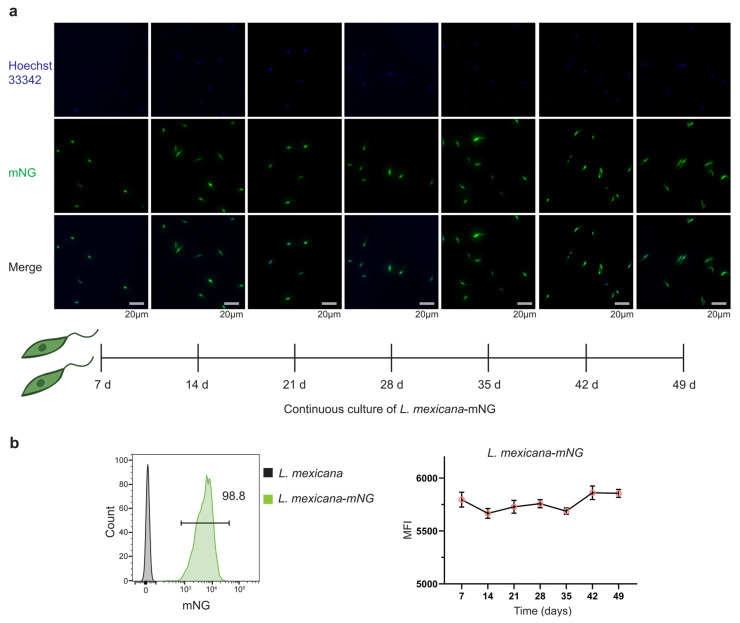
Long-term stability of mNG fluorescence in *Leishmania mexicana* during continuous in vitro culture. (**a**) Representative fluorescence images of *L. mexicana-mNG* parasites maintained under continuous culture for 49 days (7 weeks) without antibiotic selection are shown. Nuclei and kinetoplasts were counterstained with Hoechst 33342 (blue). mNG fluorescence (green) remained strong and homogeneous throughout the culture period from three independent experiments. Scale bar, 20 μm. (**b**) Representative flow cytometry histograms of *L. mexicana-mNG* promastigotes after 49 days of continuous culture in the absence of antibiotic selection. Compared with the WT control, 98.8% of the population retained strong mNG-positive fluorescence. Kinetic analysis of MFI over the 49-day culture period. MFI remained stable, fluctuating between 5500 and 6000 arbitrary units, indicating sustained reporter expression under non-selective conditions. Data are presented as mean ± SD from three independent experiments.

To assess fluorescence robustness and stability of *L. mexicana-mNG* reporter line, we focused on its expression across parasite stages and during prolonged culture without selection. Our mNG-based reporter system yielded high-contrast visualization in mid-log-phase promastigotes, with the fluorescent signal filling the cytoplasm and flagellum uniformly. The distinct outlines observed under confocal microscopy aligned perfectly with the spindle-shaped morphology seen in brightfield, confirming the reliability of the labeling. To assess signal stability, parasites were maintained in continuous culture for 49 days in the absence of selective antibiotics, and longitudinal fluorescence imaging combined with Hoechst 33342 nuclear staining demonstrated uniform and persistent mNG expression across the population over time. Together, these results indicate that integration of the mNG-FLAG cassette at the 18S rRNA locus supports stable, non-perturbing reporter expression suitable for long-term cell biological analyses (Figure 2a).

To quantitatively assess the population-level stability of the *L. mexicana-mNG* reporter generated by targeted integration at the 18S rRNA locus, parasites were analyzed by flow cytometry following prolonged culture. After 49 days (~7 weeks) of cultivation in the absence of selective antibiotics, 98.8% of the transgenic population retained mNG-positive fluorescence, indicating minimal loss of the integrated reporter cassette during long-term proliferation (Figure 2b). In parallel, expression stability at the single-cell level was evaluated by monitoring MFI over time. From day 7 to day 49, the MFI remained highly consistent, ranging between approximately 5500 and 6000 relative fluorescence units with minimal fluctuation (Figure 2b). Together, these data demonstrate that genomic integration at the high-copy 18S rRNA locus supports durable, homogeneous reporter expression during extended in vitro propagation.

**Figure 3:**
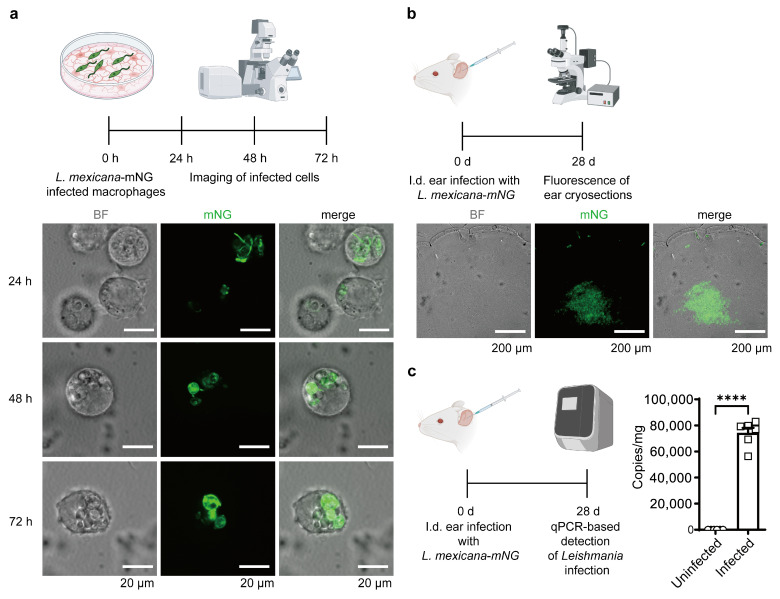
Validation of the mNG reporter system in macrophage infection and murine models. (**a**) Validation in an in vitro macrophage infection model. Schematic representation (**left**) and representative confocal images (**right**) of RAW 264.7 macrophages infected with *L. mexicana-mNG*. Merged brightfield (BF) and fluorescence images at 24, 48, and 72 h post-infection (hpi) confirm the robust and sustained mNG signal within host parasitophorous vacuoles, demonstrating the stability of the reporter during promastigote-to-amastigote differentiation. Scale bars = 20 μm. (**b**) In vivo validation in a murine cutaneous leishmaniasis model. Schematic of intradermal (i.d.) infection in BALB/c mouse ears (*n* = 6). At 28 days post-infection (dpi), fluorescence microscopy of ear cryosections reveals dense clusters of mNG-positive parasites within dermal lesions, indicating successful tracking of chronic amastigote-stage infection in vivo. Scale bars = 200 μm. (**c**) Molecular quantification of parasite burden. qPCR-based detection of *Leishmania* genomic DNA (Hsp70 gene) in ear tissues at 28 dpi. The parasite load is expressed as Hsp70 copy numbers per mg of tissue DNA. Data are presented as mean ± SD. Statistical significance was determined by Student’s *t*-test (****, *p* < 0.0001).

To evaluate whether constitutive expression of the mNG reporter affects parasite infectivity and to demonstrate its experimental utility, macrophages were infected with *L. mexicana-mNG* and monitored over an extended duration. Confocal imaging at 24, 48, and 72 h post-infection revealed robust and consistent green fluorescence within the host parasitophorous vacuoles. The mNG signal remained highly stable throughout the promastigote-to-amastigote differentiation and subsequent intracellular replication, while host cell morphology remained unaltered. This persistent signal provided superior contrast against the host cell background, facilitating clear distinction of parasites without the need for additional staining. These observations indicate that integration of the mNG-FLAG cassette does not impair parasite internalization, early survival, or long-term intracellular persistence. Together, these findings demonstrate that *L. mexicana-mNG* combines stable, high-intensity reporter expression with preserved infective properties, establishing it as a reliable tool for long-term host–pathogen interaction analyses (Figure 3a).

To assess the performance and stability of the mNG reporter in a physiological context, BALB/c mice (*n* = 6 per group) were infected intradermally in the ear pinna with 1 × 10^6^ metacyclic promastigotes of the *L. mexicana-mNG* strain and monitored for 28 days. Fluorescence imaging of cryosectioned ear tissue at 28 dpi revealed robust and highly localized mNG signal within infected regions (Figure 3b). To confirm the authenticity of the observed fluorescence signals and accurately quantify the parasite burden, we performed qPCR analysis targeting the Hsp70 gene. In alignment with the imaging data, molecular quantification from six biological replicates (*n* = 6) revealed a high parasite load in the *L. mexicana-mNG* group compared to uninfected controls (*p* < 0.0001, Figure 3c). Notably, bright green fluorescence persisted nearly one month post-infection in the absence of antibiotic selection, demonstrating that genomic integration at the 18S rRNA locus remains stable through promastigote-to-amastigote differentiation and multiple rounds of intracellular replication in an in vivo environment. These observations indicate that *L. mexicana-mNG* provides reliable, high-intensity reporter expression in vivo, enabling precise visualization and quantification of parasite burden in a murine infection model.

### 3.3. Utility in Infection Models and Proof-of-Concept Protein Tagging via PFR2–mNG

**Figure 4:**
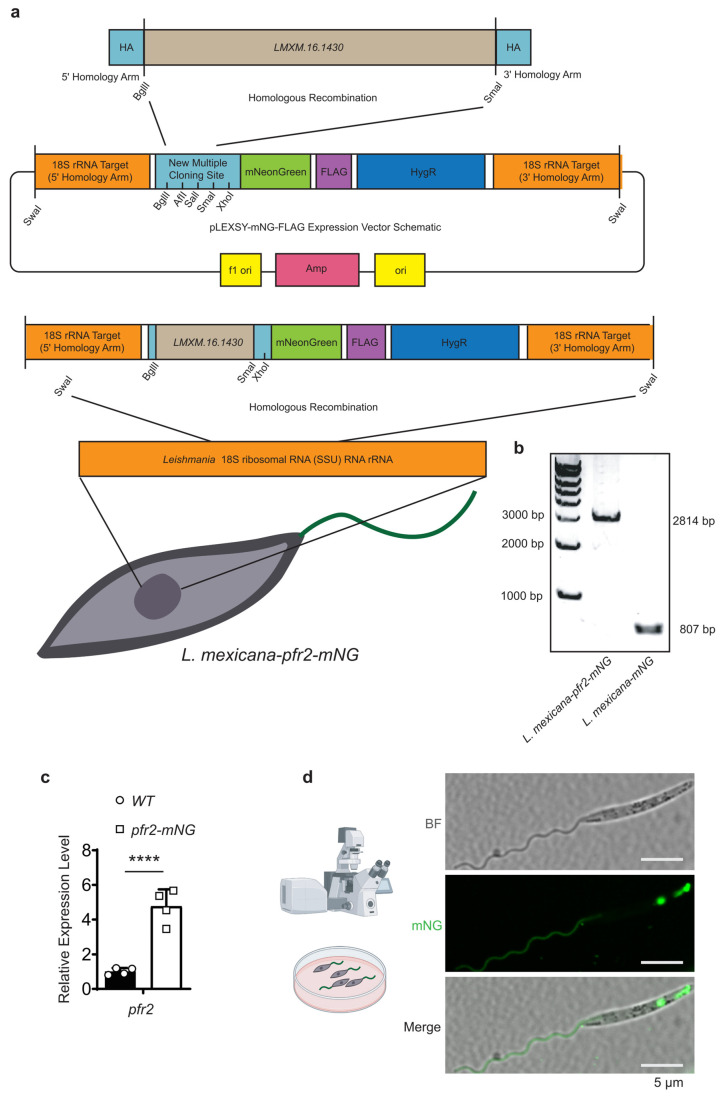
Endogenous PFR2-mNG tagging and localization in *L. mexicana*. (**a**) Schematic of the genomic integration strategy. The *pLEXSY-mNG-FLAG* vector was modified to include a multiple cloning site (MCS) enabling N-terminal fusion to mNG. The flagellar protein gene *LmxM.16.1430 (PFR2)* was cloned into the MCS, and the construct was linearized with SwaI and integrated into the 18S rRNA locus of *L. mexicana* via homologous recombination. The vector carries a hygromycin resistance cassette (HygR) for selection. (**b**) Molecular validation of site-specific integration. Diagnostic PCR of genomic DNA from transgenic parasites using integration-specific primers yielded the expected amplicons (2814 bp and 807 bp), confirming correct insertion of the *PFR2-mNG* cassette at the ssu locus. Molecular weight markers (bp) are shown on the left. (**c**) Relative mRNA expression levels of PFR2 in wild-type (WT) and pfr2-mNG integrated *L. mexicana* promastigotes. Total RNA was extracted from mid-log-phase parasites, and PFR2 transcript abundance was normalized to the endogenous GAPDH (*LmxM.30.2470*) control. Data are represented as mean ± SEM from four independent biological replicates. Statistical significance was determined by Student’s *t*-test; **** *p* < 0.0001. (**d**) Subcellular localization of the *PFR2-mNG* reporter. Confocal images of a mid-log-phase promastigote show mNG fluorescence specifically localized along the paraflagellar rod throughout the flagellum (green). BF, brightfield. Scale bar, 5 μm.

To facilitate high-level visualization of flagellar proteins and to validate the applicability of the *pLEXSY-mNG-FLAG* system for subcellular localization studies in *L. mexicana*, the gene *LmxM.16.1430 (PFR2)*, a well-characterized flagellar protein, was selected as a representative example. PFR2 was cloned into the multiple cloning site of the *pLEXSY-mNG-FLAG* platform and targeted for genomic integration at the 18S rRNA locus via homologous recombination following SwaI linearization (Figure 4a,b). This strategy ensured constitutive, site-specific expression of the *PFR2-mNG* fusion protein, providing robust fluorescence for convenient imaging while preserving endogenous localization patterns.

To evaluate the expression efficiency of our re-engineered molecular toolbox, we measured the transcript levels of the flagellar marker PFR2 in both wild-type (WT) and pfr2-mNG integrated lines. Quantitative real-time PCR (RT-qPCR) analysis revealed a significant increase in PFR2 mRNA abundance in the transgenic parasites compared to the endogenous PFR2 levels in WT cells (Figure 4c). This robust transcriptional output aligns with the strong fluorescence observed via microscopy, confirming that genomic integration into the high-copy 18S rRNA locus provides the high-level protein labeling required for high-contrast subcellular visualization.

Confocal laser scanning microscopy of mid-log-phase promastigotes revealed strong green fluorescence along the entire length of the flagellum, consistent with the established localization of PFR2 (Figure 4d). To provide a comprehensive overview of the technical performance and the specific applications of each construct developed in this study, we have summarized the vector configurations, their respective subcellular targets, and the corresponding experimental validation outcomes in Table 1. This systematic comparison highlights the versatility of the 18S rRNA-targeted platform, showing that it maintains high-contrast labeling and robust transcriptional output across different biological contexts, from stable promastigote cultures to long-term in vivo infections.

In addition to the predominant flagellar signal, cytoplasmic fluorescence was also observed, with enrichment in the perinuclear region, consistent with newly synthesized PFR2-mNG undergoing assembly and intracellular trafficking prior to incorporation into the flagellar paraflagellar rod. Brightfield imaging confirmed that transgenic parasites retained the characteristic spindle-shaped morphology and normal flagellar dimensions of wild-type *L. mexicana*. These data illustrate the feasibility of using the *pLEXSY-mNG-FLAG* system for endogenous tagging. However, as with any fusion protein, the successful labeling of PFR2 does not necessarily guarantee the functional neutrality or fluorescence stability of mNG when conjugated to other protein targets. Nevertheless, this case study demonstrates that the system can enable robust visualization of subcellular protein localization and supports downstream genetic analyses in Leishmania without perturbing cellular architecture in this specific context.

## 4. Discussion

In this study, we established a genetically stable and high-performance fluorescent reporter platform in *L. mexicana* by integrating a MCS–mNG fusion cassette into the 18S rRNA locus. This work establishes a systematic and efficient labeling platform that complements traditional techniques, providing a versatile and robust molecular toolbox for the long-term visualization of parasites across their complex life cycle [16,20].

The primary challenge in generating reporter strains in *Leishmania* is the maintenance of transgene expression without continuous antibiotic selection, especially during long-term in vivo infections. Traditional episomal vectors are prone to asymmetric segregation and rapid loss in the absence of antibiotics. Our findings that 98.8% of the population retained robust mNG fluorescence after 49 days of continuous culture without selection highlight the exceptional stability of the 18S rRNA integration. This stability is likely attributed to the high-copy nature and high transcriptional activity of the ssu locus, which ensures that the reporter gene is maintained as a stable chromosomal component within the highly active ribosomal RNA locus. Our RT-qPCR analysis further confirmed the high transcriptional efficiency of this system, demonstrating a robust abundance of reporter transcripts compared to endogenous levels (Figure 4c). Unlike recent studies utilizing CRISPR/Cas9 for endogenous tagging—which may be limited by low physiological expression levels—our approach intentionally utilizes the high transcriptional machinery of the ssu locus to achieve the enhanced brightness required for high-contrast imaging in complex host environments [21,22,23].

As a technical development, the specific localization of mNG to the paraflagellar rod (PFR) serves as a proof-of-concept for the effectiveness of our platform. The PFR is essential for flagellar motility and has been implicated in host–parasite interactions. Our observation that the mNG signal remains restricted to the flagellum in both promastigotes and the early stages of amastigote transformation within macrophages confirms that the PFR2-mNG fusion is a reliable indicator of flagellar biogenesis. This opens up possibilities for studying the mechanisms of flagellar remodeling during the transition from the motile insect stage to the intracellular mammalian stage, a process that is critical for the parasite’s survival and virulence [6,24,25].

A major highlight of our study is the robust performance of the *L. mexicana-mNG* strain in the murine cutaneous leishmaniasis model. The persistence of a high-contrast fluorescent signal at 28 days post-infection within the dermal tissue represents a notable enhancement compared with previously reported reporter strains that often suffer from fluorescence quenching or loss in the acidic environment of the phagolysosome. The brightness of mNeonGreen, combined with the stable genomic integration, enhances detection sensitivity for deep-tissue imaging, potentially enabling sensitive tracking of parasite dissemination to draining lymph nodes or visceral organs [9,26,27,28,29,30,31,32,33].

In conclusion, the re-engineered pLEXSY platform represents a refined technical framework that provides a versatile ‘plug-and-play’ tool for the *Leishmania* research community. The modular nature of our modified *pLEXSY-mNG-FLAG* vector allows for the rapid tagging of other proteins of interest. This system provides a technical foundation that facilitates high-throughput in vivo drug screening and high-resolution intravital microscopy, offering a standardized approach for future functional genomics studies [22,34].

## Figures and Tables

**Table 1:** Summary of the molecular toolbox components and experimental validation.

pLEXSY	Target Protein	Targeted Subcellular Structure	Validation Method (s)	Key Observations
pLEXSY	(MCS-mNG)	Cytosol (Constitutive)	Flow Cytometry, In vivo Imaging	Stable long-term expression; High photostability
pLEXSY-mNG	PFR2	Paraflagellar Rod (Flagellum)	RT-qPCR, Fluorescence Microscopy	High-contrast flagellar labeling; Robust transcript levels

## Data Availability

The nucleotide sequence data presented in this study have been deposited in the GenBank database under the accession numbers PZ269476 (pLEXSY-mNG-FLAG-18S) and PZ269477 (pLEXSY-LmxPFR2-mNG-FLAG). Other data supporting the findings of this study are available from the corresponding authors upon reasonable request.
